# NOX4-derived ROS-induced overexpression of FOXM1 regulates aerobic glycolysis in glioblastoma

**DOI:** 10.1186/s12885-021-08933-y

**Published:** 2021-11-05

**Authors:** Xiangsheng Su, Yihang Yang, Qing Yang, Bo Pang, Shicheng Sun, Yanjun Wang, Qiujiang Qiao, Changfa Guo, Huanting Liu, Qi Pang

**Affiliations:** 1 Department of Neurosurgery, Shandong Provincial Hospital, Cheeloo College of Medicine, Shandong University, Jinan, 250012 Shandong China; 2 Department of Neurosurgery, Shandong Provincial Hospital Affiliated to Shandong First Medical University, Jinan, 250012 Shandong China; 3 Department of Radiation Oncology, The First Affiliated Hospital of Guangxi Medical University, Nanning, 530022 Guangxi China; 4 Department of Neurosurgery, Qilu Hospital, Cheeloo College of Medicine, Shandong University, Jinan, 250012 Shandong China; 5 Department of Neurosurgery, Linyi People’s Hospital, Cheeloo College of Medicine, Shandong University, Linyi, 276003 Shandong China

**Keywords:** NOX4, ROS, FOXM1, Aerobic glycolysis, Glioblastoma

## Abstract

**Background:**

Increased expression of the transcription factor Forkhead box M1 (FOXM1) has been reported to play an important role in the progression and development of multiple tumors, but the molecular mechanisms that regulate FOXM1 expression remain unknown, and the role of FOXM1 in aerobic glycolysis is still not clear.

**Methods:**

The expression of FOXM1 and NADPH oxidase 4 (NOX4) in normal brain tissues and glioma was detected in data from the TCGA database and in our specimens. The effect of NOX4 on the expression of FOXM1 was determined by Western blot, qPCR, reactive oxygen species (ROS) production assays, and luciferase assays. The functions of NOX4 and FOXM1 in aerobic glycolysis in glioblastoma cells were determined by a series of experiments, such as Western blot, extracellular acidification rate (ECAR), lactate production, and intracellular ATP level assays. A xenograft mouse model was established to test our findings in vivo.

**Results:**

The expression of FOXM1 and NOX4 was increased in glioma specimens compared with normal brain tissues and correlated with poor clinical outcomes. Aberrant mitochondrial reactive oxygen species (ROS) generation of NOX4 induced FOXM1 expression. Mechanistic studies demonstrated that NOX4-derived MitoROS exert their regulatory role on FOXM1 by mediating hypoxia-inducible factor 1α (HIF-1α) stabilization. Further research showed that NOX4-derived MitoROS-induced HIF-1α directly activates the transcription of FOXM1 and results in increased FOXM1 expression. Overexpression of NOX4 or FOXM1 promoted aerobic glycolysis, whereas knockdown of NOX4 or FOXM1 significantly suppressed aerobic glycolysis, in glioblastoma cells. NOX4-induced aerobic glycolysis was dependent on elevated FOXM1 expression, as FOXM1 knockdown abolished NOX4-induced aerobic glycolysis in glioblastoma cells both in vitro and in vivo.

**Conclusion:**

Increased expression of FOXM1 induced by NOX4-derived MitoROS plays a pivotal role in aerobic glycolysis, and our findings suggest that inhibition of NOX4-FOXM1 signaling may present a potential therapeutic target for glioblastoma treatment.

**Supplementary Information:**

The online version contains supplementary material available at 10.1186/s12885-021-08933-y.

## Background

Glioblastoma multiforme (GBM) is the most frequent primary malignant brain tumor in adults, with a median overall survival of less than 2 years [1]. There are a variety of available treatments, such as surgery, chemotherapy, and radiation; however, because it represents a highly heterogeneous group of neoplasms, GBM is still an incurable disease [2]. Therefore, elucidating the intrinsic molecular mechanisms underlying the abnormal features of GBM is pivotal for the identification of novel targets.

Aerobic glycolysis, or the Warburg effect, a recognized hallmark of cancer, is the process of oxidation of glucose into pyruvate followed by lactate production rather than oxidative phosphorylation under physiological oxygen conditions [3]. In recent years, altering metabolic gene expression in glycolysis has become widely regarded as a desirable target for cancer therapeutics. For example, blocking glucose transporter 1 (GLUT1) with the small-molecule inhibitor WZB117 downregulates glycolysis and inhibits cancer cell growth in vitro and in vivo [4]. Targeting of lactate dehydrogenase A (LDHA) and related metabolic pathways offers efficacious strategies for cancer cell treatment [5]. Hexokinase 2 (HK2) depletion inhibits tumor growth and increases sensitivity to cell death inducers such as radiation and temozolomide [6]. However, selective blockade of these glycolysis-related enzymes in cancer cells comes with side effects, which remains a critical challenge because these enzymes are ubiquitously expressed in all mammalian cells [7]. Therefore, the identification of initial oncogenic signaling is vital for the development of glycolysis inhibition strategies.

Oxidative stress has been shown to play a major role in aerobic glycolysis and to lead to cancer development and progression. Increased reactive oxygen species (ROS) production serves a critical signaling function under physiological conditions [8]. NADPH oxidase 4 (NOX4) is a NOX family isoform that constitutively produces ROS and is involved in multiple biological functions during cancer progression [8]. The majority of existing evidence demonstrates that increased expression of NOX4 and NOX4-induced ROS production play important roles in aerobic glycolysis [9, 10]. NOX4-derived ROS often act as secondary signaling molecules affecting the expression or stabilization of downstream genes, such as hypoxia-inducible factor1α (HIF-1α) [10]. Nevertheless, the comprehensive mechanisms underlying the elevated levels of NOX4-derived ROS in aerobic glycolysis and downstream target genes in glioblastoma cells remain largely unknown. Recently, Forkhead box M1 (FOXM1), a member of the Forkhead box (FOX) transcription factor family, was recognized as a master regulator of progression in a variety of cancer cell types [11, 12]. FOXM1 is frequently upregulated in the majority of human solid cancers and is highly associated with poor clinical prognosis, including but not limited to hepatocellular carcinoma and lung adenocarcinoma [13, 14]. Recent studies have revealed that increased FOXM1 expression regulates the transcription of target genes that contribute to cell proliferation, cell cycle progression, and metabolic reprogramming [13, 15, 16]. In brief, these observations demonstrate a strong reliance of tumor cells on FOXM1. Therefore, it is urgent to investigate the mechanisms that regulate FOXM1 expression. To date, whether FOXM1 is regulated by NOX4-derived ROS and the role of FOXM1 expression in glioblastoma aerobic glycolysis remain unknown.

In the present study, we found that FOXM1 and NOX4 were overexpressed in glioblastoma and established these factors as strong biomarkers for diagnosis and prognosis. Our data demonstrated that overexpression of NOX4, a producer of mitochondrial ROS that stabilizes HIF-1α, induced aerobic glycolysis by regulating FOXM1 expression. Taken together, these findings indicate that targeting NOX4-FOXM1 signaling may represent a novel therapeutic strategy for the treatment of glioblastoma.

## Methods

### Cell lines and cell culture

Human glioblastoma cell lines (U87MG, A172, U251, LN229, T98G, and U373) and a normal human astroglia (NHA) cell line were obtained from the Cell Bank of Type Culture Collection of the Chinese Academy of Sciences and were cultured in Dulbecco’s modified Eagle’s medium (DMEM, Gibco, USA) containing fetal bovine serum (FBS) at a final concentration of 10% at 37 °C with 5% CO_2_ in humidified incubators. For in vitro hypoxia experiments, cells were cultured under consistent 1% O_2_ hypoxic conditions.

### Treatment with N-acetyl cysteine (NAC), hydrogen peroxide (H_2_O_2_), and Tempol

To test the effects of N-acetyl cysteine (NAC; HY-B0215, MCE, USA), H_2_O_2_ (7722-84-1, Sigma, USA), and Tempol (HY-100561, MCE, USA) on glioblastoma cells, compounds were dissolved and stored according to the manufacturer’s instructions. Then, the compounds were diluted in complete DMEM, and glioblastoma cells were exposed to NAC, H_2_O_2_, or Tempol at 37 °C for 24 h in complete medium. The final concentrations were 0, 0.1, 0.5, 1, 5, and 10 mM NAC; 0, 0.1, 0.5, 1, 5, and 10 μM H_2_O_2_; and 1 mM Tempol.

### The Cancer genome atlas (TCGA) dataset analysis

Normalized gene-level RNA-seq and corresponding clinical data of 5 normal human brain tissue samples, 529 LGG cancer tissue samples, and 169 GBM cancer tissue samples from patients were selected from the most recent update of The Cancer Genome Atlas (TCGA) database (https://cancergenome.nih.gov/) according to the parameters described. Quantile normalization was used to normalize mRNA expression [transcription fragments per million base pairs per thousand base fragments (FPKM)] data. Low-grade glioma (LGG) was defined as WHO grade I-III, and glioblastoma multiforme (GBM) was defined as WHO IV according to the World Health Organization (WHO) classification of central nervous system tumors.

### Clinical human specimens and immunohistochemical (IHC) staining

Normal brain tissue samples (*n* = 6) were collected from patients who needed surgical treatment for severe epilepsy or underwent craniocerebral decompression treatment for brain trauma. Glioma samples, including 6 WHO II tissues, 6 WHO III tissues, and 15 GBM tissues, were collected from patients who underwent glioma resection surgery. All samples were obtained from the Department of Neurosurgery of Shandong Provincial Hospital. This work was approved by the ethics committee of Shandong Provincial Hospital. Immunohistochemical staining of paraffin-embedded tissues was performed with antibodies against NOX4 (1:200, ab13303, Abcam, USA) and FOXM1 (1:100, 13,147–1-AP, Proteintech, China) to detect protein expression according to standard IHC procedures.

### Measurement of ROS

Cells were plated in 24-well plates and loaded with 5 μM MitoSOX Red (M36008, Invitrogen, USA) in phenol-free DMEM for 10 min at 37 °C with 5% CO_2_ and washed with PBS. MitoSOX red fluorescence intensity was determined at 510 nm excitation and 580 nm emission under an ImageXpress Microconfocal microscope (Molecular Devices, USA).

### Immunofluorescence

Cells were seeded and grown on 24-well plates and stained with MitoTracker Deep Red (1:5000, BB-44113, BestBio, China) for 30 min. Then, the cells were fixed with 4% paraformaldehyde for 20 min and permeabilized with 0.5% Triton X-100 for 10 min. The cells were blocked for 30 min and incubated with NOX4 primary antibodies (1:200, ab13303, Abcam, USA) at room temperature for 1 h. FITC-conjugated secondary antibodies (1:500, ab150077, Abcam, USA) were used to detect the primary antibodies, and the cells were mounted in an anti-fade reagent with DAPI and visualized on an Olympus fluorescence microscope.

### Western blot

Standard Western blotting was carried out using whole-cell protein lysates as described in our previous study [17]. Cells or tissues were lysed in RIPA buffer. The protein concentrations were normalized with a BCA assay kit (Thermo Fisher Scientific, Carlsbad, CA, USA). After centrifugation, 20 μg of cell or tissue lysate was electrophoresed in sodium dodecyl sulfate (SDS)-polyacrylamide gel (10% or 12%) and transferred to polyvinylidene fluoride (PVDF) membranes. After blocking with 5% skimmed milk in TBST (Tris-buffered saline with 0.1% Tween-20) at room temperature for 2 h, the membranes were incubated overnight at 4 °C with primary antibodies against NOX4 (1:2000, ab13303, Abcam, USA), FOXM1 (1:1000, 13,147–1-AP, Proteintech, China), HIF-1α (1:1000, 20,960–1-AP, Proteintech, China), PLK1 (1 μg/ml, ab17057, Abcam, USA), Cyclin B1 (1:2000, ab181593, Abcam, USA), LDHA (1:1000, ab101562, Abcam, USA), GLUT1 (1:5000, ab115730, Abcam, USA), HK2 (1:5000, 2867, CST, USA), and β-actin (1:5000, 66,009–1-Ig, Proteintech, China) as a control. After washing with TBST, the membranes were incubated with secondary antibodies (anti-rabbit IgG, ZB-2301, and anti-mouse IgG, ZB-2305, ZSGB-Bio, China) diluted in blocking buffer for 1 h at room temperature. The protein levels were quantified by densitometry using an Amersham Imager 680 (GE Healthcare, USA). Unprocessed images of the immunoblots are provided in the Supplementary Materials.

### RNA isolation and real-time quantitative PCR (qPCR)

Total RNA was extracted from tissues or cells using TRIzol reagent according to the instructions of the manufacturer (Invitrogen). The RNA purity was assessed by spectrophotometry (absorbance at 260 nm A260/A280 > 1.8). Approximately 1000 ng of total isolated RNA was subsequently transcribed into cDNA using a high-capacity cDNA reverse transcription kit (Vazyme, China) according to the manufacturer’s instructions. qPCR was then performed for 1 min at 95 °C using a SYBR Green kit (Qiagen, Germany) on a Roche LightCycler 480 system with 200 nM primers, followed by amplification in 40 cycles of 95 °C for 15 s, 60 °C for 15 s, and 72 °C for 20 s. Relative expression was normalized to β-actin using quantification as 2^−ΔΔCT^ (comparative threshold cycle). The primer sequences used were as follows: β-actin (forward: 5′-CACCATTGGCAATGAGCGGTTC-3′; reverse: 5′-AGGTCTTTGCGGATGTCCACGT-3′) and FOXM1 (forward: 5′-GGCCATCCCCAACAATGCTA-3′; reverse: 5′-AGGTCTCCAGGGTCACTTCT-3′).

### Measurement of extracellular acidification rate (ECAR)

Cells were collected and plated in Agilent Seahorse XFe96 plates at a density of 5 × 10^4^ cells per well and allowed to adhere for 4 h in a standard incubator. Cells were next equilibrated with XF Base media at 37 °C for 1 h in an incubator lacking CO_2_ and then serum-starved for 1 h in glucose-free media-containing treatments. Measurement of ECAR was performed by the Glycolytic Stress Test Kit (103020–100, Agilent Technologies, USA). Briefly, cells were subjected to sequential addition of glucose (10 mM), oligomycin (1.0 μM), and 2-DG (50 nM) according to the XF Glycolysis Stress Test protocol on a Seahorse XFe96 Extracellular Flux Analyzer (Agilent Technologies, USA). Data were analyzed using Wave software.

### Measurement of lactate production

Cells were seeded and cultured in DMEM for 24 h. The culture medium was collected to measure the lactate concentration using a lactate assay kit (CAT#A019–2-1, Jiancheng, China), and the values were normalized to the protein concentration.

### Measurement of ATP production

ATP levels were measured using an ATP assay kit (Cat#S0026, Beyotime, China) according to the manufacturer’s instructions. Luminescence was measured using a luminescence reader, and the values were normalized to the protein concentration.

### Cell transfection

Full-length NOX4, HIF-1α, and FOXM1 cDNA sequences were cloned into the pcDNA3.1-EGFP overexpression vector (GenePharm, Shanghai, China) to generate stable overexpression clones, using empty vector (EV) as the negative control. Lentiviruses stably expressing nontargeting control (NTC), shNOX4, and shFOXM1 were also purchased from GenePharm. The nucleotide sequences of the shRNAs were as follows: NTC: 5′-TTCTCCGAACGTGTCACGT-3′; shNOX4, 5′-GGTATATCCGGAGCAATAAGC-3′; shFOXM1, 5′-GCTGGGATCAAGATTATTA-3′. After infection according to the manufacturer’s instructions for 24 h, glioblastoma cells were treated with 5 μg/ml puromycin for 96 h. Control siRNA (siRNA sense: 5′-UUCUCCGAACGUGUCACGUTT-3′, antisense: 5′- ACGUGACACGUUCGGAGAATT-3′ and HIF-1α siRNA (siRNA sense: 5′- CUGAUGACCAGCAACUUGA-3′, antisense: 5′- UCAAGUUGCUGGUCAUCAG-3′) were synthesized by GenePharma, and siRNA transfection was performed with Lipofectamine RNAiMAX (Invitrogen, USA) according to the manufacturer’s instructions. Transfection efficiency was confirmed as positive by Western blot.

### Luciferase reporter assay

The wild-type (WT) FOXM1 promoter construct or the mutated (MUT) FOXM1 promoter construct was transfected into U87MG glioblastoma cells with pGL4.2-basic-Luc reporter plasmids according to the manufacturer’s instructions and then cotransfected with empty vector or HIF-1α overexpression plasmid and pRL-TK for 48 h. The relative reporter gene activity was determined using the Dual-Luciferase Assay System (Promega; USA) according to the manufacturer’s protocol. pRL-TK encoding Renilla luciferase was used as an internal control to assess transfection efficiency. All plasmids above were sequenced by GenePharm (Shanghai, China).

### Cell counting kit (CCK)-8 assays

Cell viability was evaluated with Cell Counting Kit (CCK)-8 assays (GK10001, Glpbio, USA) according to the manufacturer’s instructions. Briefly, the indicated glioblastoma cells were seeded at a density of 5 × 10^3^ cells/well in 96-well culture plates, and each sample was seeded in three replicates. CCK-8 solution was added to each well at a final concentration of 10% and incubated for 1 h. The optical density (OD) absorbance of each well was measured at 450 nm using a Thermo Scientific microplate reader to determine cell viability.

### EdU proliferation assays

The proliferation assay was performed by EdU (5-ethynyl-2′-deoxyuridine) to measure cell proliferation. In brief, the indicated cells were seeded in 96-well plates (2 × 10^4^ cells/well) and cultured overnight. Subsequently, the cells were washed three times with PBS and then incubated in serum-free DMEM containing 50 μM EdU (RiboBio, China) for 2 h. Then, the cells were fixed with 4% polyformaldehyde in PBS for 30 min at room temperature. Finally, cells were incubated with Apollo staining solution and Hoechst 33342 for 30 min. The proliferation index was defined as the percentage of EdU-positive cells relative to the total cells.

### Animal studies

Approximately 1 × 10^5^ U87MG cells, including EV + NTC, NOX4 + NTC, and NOX4+ shFOXM1 cell groups, were intracranially injected into female pathogen-free athymic BALB/c nude mice obtained from Vital River Laboratories (*n* = 6 mice per group). At 5 weeks after implantation, tumors were harvested for HE staining, ATP measurement, immunohistochemistry, and Western blot assays. All animal studies were conducted with approval from the ethics committee of Shandong Provincial Hospital.

### Statistical analysis

All experiments were carried out at least three times. For measurement of ROS, immunofluorescence staining, IHC, EdU assay, Western blot assay, and HE assay, representative images are shown. The results were presented as the mean ± S.D. of at least three independent experiments after analysis by Student’s t-test or one-way ANOVA using GraphPad Prism 7.00 (GraphPad, La Jolla, CA, USA). Relative gene expression was analyzed using the 2^−ΔΔCt^ method. The difference in FOXM1 and NOX4 expression between normal brain tissues, LGG, and GBM from the TCGA database was determined by the Wilcoxon signed-rank test. The correlations between FOXM1 expression and NOX4 expression were analyzed with Pearson’s correlation analysis. Survival analyses were performed using the Kaplan–Meier method and assessed using the log-rank test. All statistical tests were two-sided, and *P* values < 0.05 were considered significant. ns, not significant; *, *P* < 0.05; **, *P* < 0.01; ***, *P* < 0.001.

## Results

### FOXM1 is high expressed in glioma

**Fig. 1:**
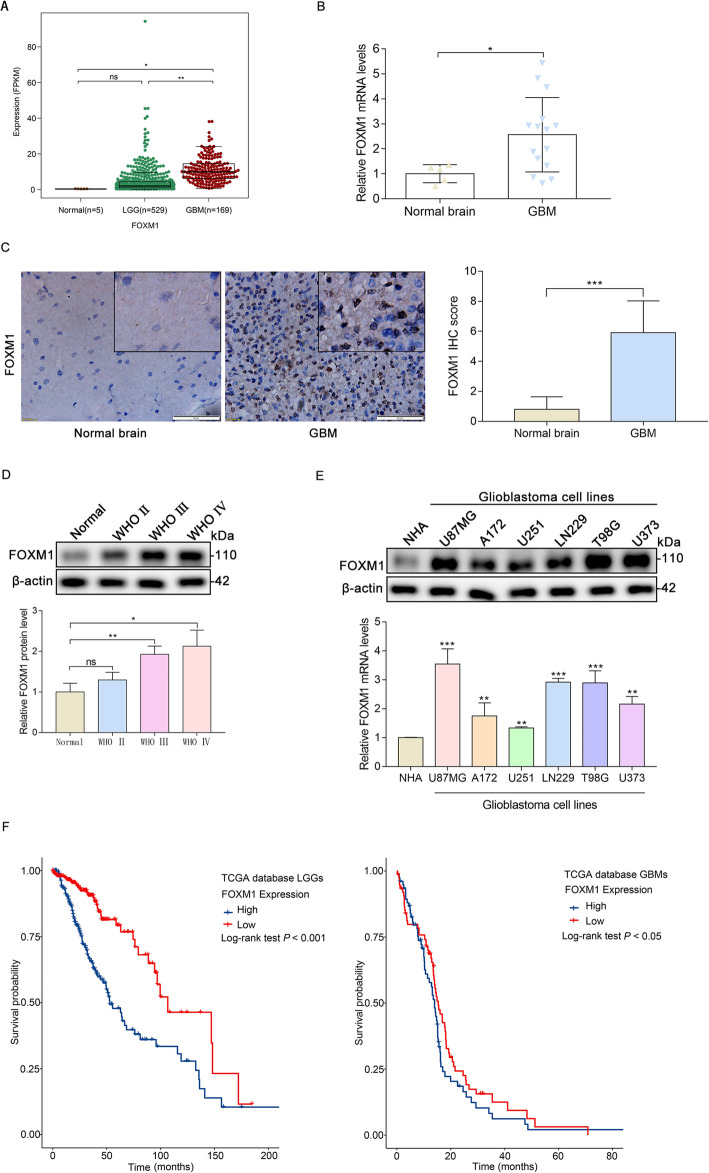
FOXM1 is high expressed in glioma. **A** Analysis of FOXM1 expression in normal brain and glioma tissues based on data from the TCGA database. **B** qPCR detection of FOXM1 mRNA expression in normal brain and glioma tissues from clinical specimens. **C** IHC analysis of FOXM1 protein expression in normal brain and GBM samples. Scale bars, 20 μm. **D** Western blot analysis of FOXM1 protein expression in normal brain and glioma with different grades. E Western blot and qPCR detection of FOXM1 expression in multiple glioblastoma cells and in normal human astroglia (NHA) cell lines. F The prognostic value of FOXM1 expression in LGG and GBM was analyzed in TCGA datasets. ns, not significant; *, *P* < 0.05; **, *P* < 0.01; ***, *P* < 0.001

To confirm the function of FOXM1 in the development of glioma, we investigated the expression of FOXM1 in normal brain and glioma specimens with different grades. We observed that compared to that in the normal brain and LGG tissues, FOXM1 mRNA expression was significantly upregulated in the GBM tissues in the TCGA database (Fig. 1a). To validate the TCGA results, we checked the mRNA and protein expression levels of FOXM1 in normal brain tissues and human glioma samples. As demonstrated, FOXM1 was also obviously highly expressed in GBM, as detected by qPCR (Fig. 1b), IHC (Fig. 1c), and Western blot (Fig. 1d). In addition, FOXM1 protein and mRNA expression levels were dramatically increased in glioblastoma cell lines compared with the normal human astroglia (NHA) cell line (Fig. 1e). FOXM1 expression levels were also related to the prognosis of glioma patients in the TCGA database. We showed that glioma patients with high FOXM1 expression had a worse prognosis than patients with low FOXM1 expression in the TCGA database (Fig. 1f). Thus, these results suggest that FOXM1 is associated with glioma progression and may serve as an independent prognostic biomarker for glioma.

### NOX4 stimulates FOXM1 expression by increasing mitochondrial ROS

**Fig. 2:**
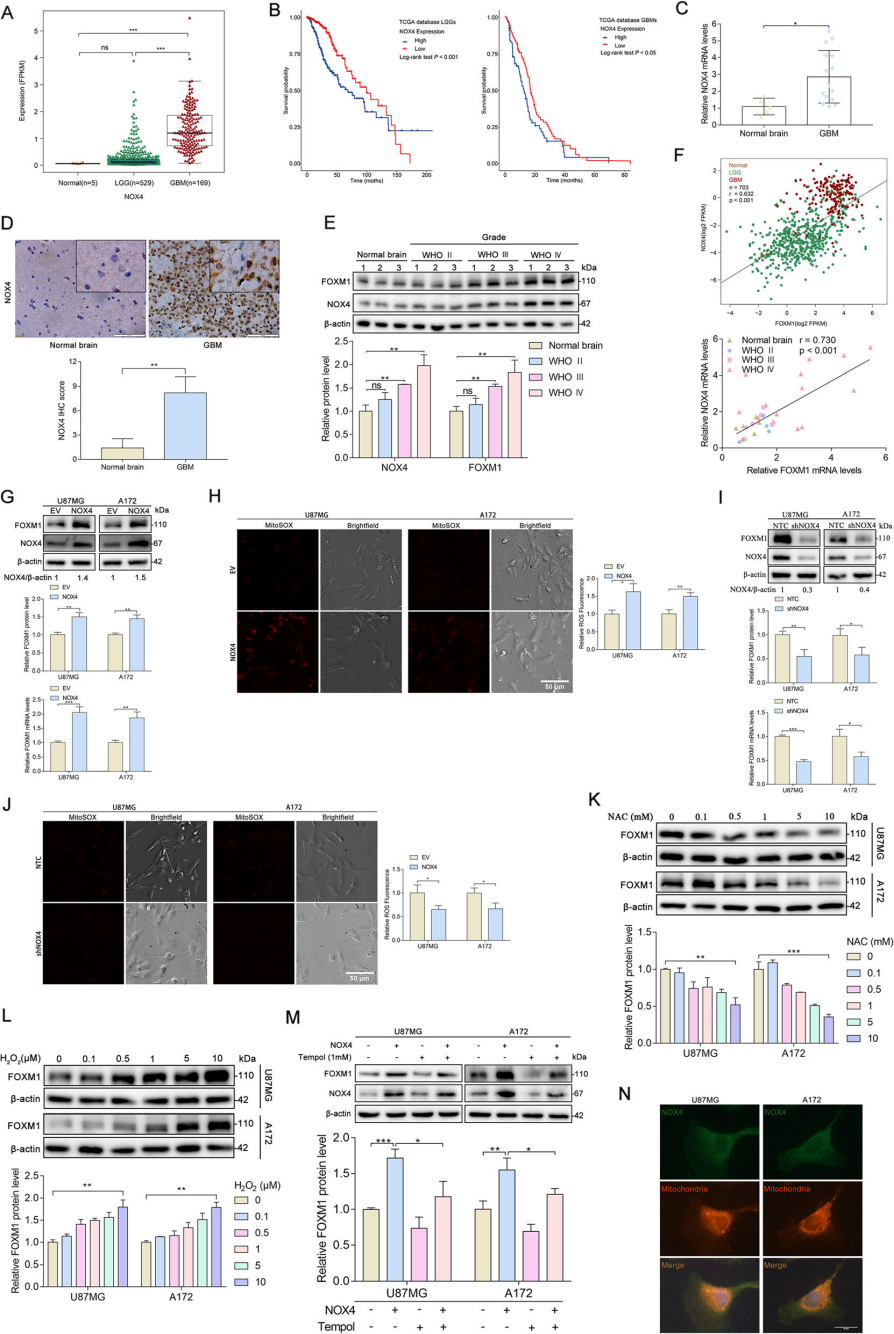
NOX4 stimulates FOXM1 expression by increasing mitochondrial ROS. **A** Analysis of NOX4 expression in normal brain and glioma tissues in TCGA datasets. **B** The prognostic value of NOX4 expression in LGG and GBM was analyzed in TCGA datasets. **C** qPCR detection of NOX4 mRNA expression in normal brain and glioma clinical specimens. **D** IHC analysis of NOX4 protein expression in normal brain and GBM. Scale bars, 20 μm. **E** Western blot analysis of NOX4 and FOXM1 protein expression in normal brain and glioma tissues of different grades. **F** The correlation between NOX4 expression and FOXM1 expression in normal brain and glioma patients according to the TCGA and our clinical samples. **G** Western blot and qPCR analysis of FOXM1 protein and mRNA levels in control or NOX4-overexpressing glioblastoma cells. **H** MitoSOX staining showing the upregulation of NOX4 affected the production of ROS in glioblastoma cells. Scale bars, 50 μm. **I** Western blot and qPCR analysis of FOXM1 protein and mRNA levels in control or NOX4 knockdown glioblastoma cells. **J** MitoSOX staining showed that the downregulation of NOX4 affected the production of ROS in glioblastoma cells. Scale bars, 50 μm. **K** Western blot analysis showing the protein expression of FOXM1 in glioblastoma cells exposed to NAC at different concentrations for 24 h. **L** Western blot analysis showing the protein expression of FOXM1 in glioblastoma cells exposed to H_2_O_2_ at different concentrations for 24 h. **M** Western blot analysis showing the protein expression of FOXM1 in glioblastoma cells treated with Mito-Tempo (1 mM) in the control or NOX4-overexpressing cells. **N** Immunofluorescence staining showing the subcellular localization of NOX4 in glioblastoma cells; the merge in yellow shows colocalization. Scale bar 50 μM. *, *P* < 0.05; **, *P* < 0.01; ***, *P* < 0.001

A moderate increase in ROS has been implicated in enhanced cell proliferation, increased cellular growth, cell survival, and cancer development [8]. Since NOX4 is thought to be a source of intracellular ROS in several types of cancer [8] and FOXM1 is aberrantly expressed in glioma, we next sought to determine the relationship between NOX4 and FOXM1. As suggested by the TCGA database analysis, NOX4 was highly expressed in glioma (Fig. 2a), and the upregulation of NOX4 indicated a shorter survival (Fig. 2b). Next, we performed qPCR, IHC, and Western blotting to detect NOX4 expression in glioma tissues. Our results showed that NOX4 was highly expressed in glioma tissues (Fig. 2c-e). Relationship analysis indicated that NOX4 and FOXM1 expression are correlated with each other in glioma (Fig. 2f). To determine the role of NOX4 regulation of FOXM1, NOX4 overexpression (NOX4) and NOX4 knockdown (shNOX4) glioblastoma cell lines were established. As expected, our results indicated that FOXM1 expression was significantly increased in NOX4-overexpressing cells compared with control cells (Fig. 2g), which coincided with increased mitochondrial ROS levels (Fig. 2h). In contrast, we found that knockdown of NOX4 expression significantly decreased the level of FOXM1 (Fig. 2i), which also coincided with decreased mitochondrial ROS production (Fig. 2j). Next, we examined whether ROS were critical for the expression of FOXM1. We found that treatment with the ROS scavenger N-acetyl-cysteine (NAC) reduced the expression of FOXM1 (Fig. 2k). To further validate the ROS dependence of FOXM1 expression, we treated cells with hydrogen peroxide (H_2_O_2_). Treatment with hydrogen peroxide also gradually increased the expression of FOXM1 in a concentration-dependent manner (Fig. 2l). Subsequently, to explore whether NOX4-derived mitochondrial ROS are involved in the induction of FOXM1 expression, Mito-Tempo (1 mM), a mitochondria-targeted superoxide dismutase mimetic with superoxide, was used. FOXM1 expression in NOX4-overexpressing cells decreased after treatment with Mito-Tempo (Fig. 2m). Finally, we investigated the subcellular localization of NOX4 in glioblastoma cells. Immunofluorescence demonstrated that NOX4 was expressed in the cytoplasm and suggested that most NOX4 localized to the mitochondria (Fig. 2n). Together, these findings reveal that NOX4-derived ROS, especially from mitochondria, are required for an increase in FOXM1 levels in glioblastoma cells.

### NOX4 positively regulates FOXM1 expression by mediating HIF-1α stabilization

**Fig. 3:**
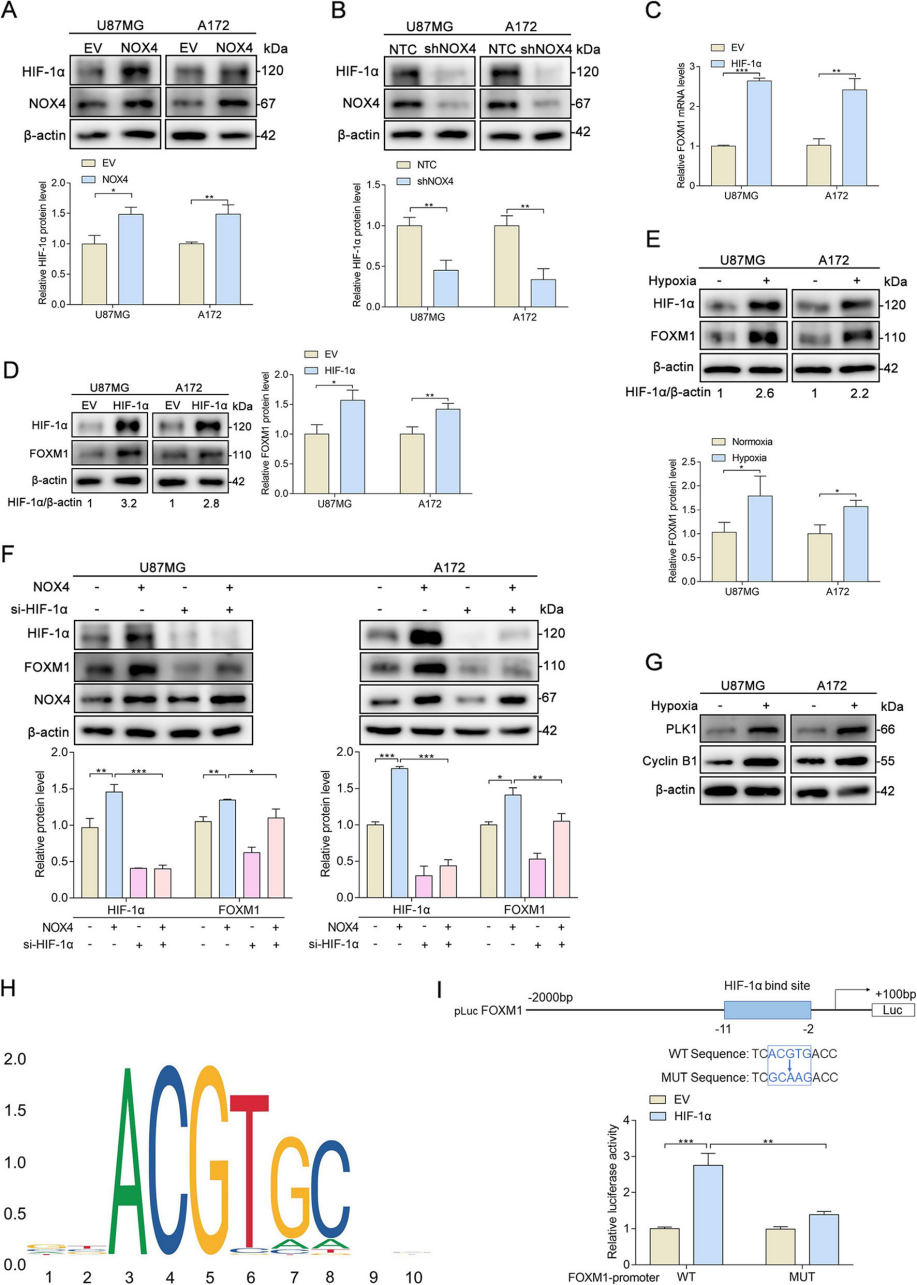
NOX4 positively regulates FOXM1 expression by mediating HIF-1α stabilization. **A** Western blot analysis showing HIF-1α protein levels in NOX4-overexpressing or NOX4-knockdown glioblastoma cells. **B** Western blot analysis showing HIF-1α protein levels in NOX4-knockdown glioblastoma cells. **C** qPCR detection of FOXM1 mRNA levels in HIF-1α-overexpressing glioblastoma cells. **D** Western blot analysis showing the FOXM1 protein levels in HIF-1α-overexpressing glioblastoma cells. **E** Western blot analysis showing the FOXM1 protein levels in normoxic and hypoxic glioblastoma cells. **F** The indicated glioblastoma cells were transfected with HIF-1α siRNA or negative control siRNA, and the protein levels of FOXM1, HIF-1α, and NOX4 were measured by Western blot. **G** Western blot analysis showing the protein levels of the FOXM1 downstream targets cyclin B1 and PLK1 in normoxic and hypoxic glioblastoma cells. **H** Map of the HIF-1α binding site sequence from the JASPAR database. **I** Sequences and positions of putative HIF-1α-binding regions on the FOXM1 promoter and luciferase reporter constructs for wild-type or mutant FOXM1 promoter were cotransfected into U87MG cells along with EV- or HIF-1α-overexpressing plasmids for 48 h. Promoter activity was examined with a dual-luciferase reporter assay kit. *, *P* < 0.05; **, *P* < 0.01; ***, *P* < 0.001

We then evaluated the potential mechanisms responsible for the expression of FOXM1 in glioblastoma cells. Since a significant increase in FOXM1 mRNA levels was observed in NOX4-overexpressing cells (Fig. 2g), while NOX4 knockdown reversed this effect (Fig. 2i), we hypothesized that NOX4 may regulate FOXM1 expression at the transcriptional level. Because elevated ROS levels contribute to the stabilization of HIF-1α [18] and NOX4 is a source of ROS production in our results, we examined the effect of NOX4 on the protein expression of HIF-1α. As expected, Western blot analysis demonstrated that in glioblastoma cells, overexpression of NOX4 induced HIF-1α protein expression (Fig. 3a), whereas the opposite was true upon NOX4 knockdown (Fig. 3b). Hypoxia-inducible factors are well known to function as master regulators of multiple genes that contribute to cancer progression [19]. HIF-1α overexpression obviously and dramatically increased FOXM1 mRNA levels (Fig. 3c) and protein levels (Fig. 3d). We also extended our analysis to hypoxic conditions by detecting FOXM1 expression in glioblastoma cells exposed to severe hypoxia or normoxia for 48 h. FOXM1 expression was markedly increased in glioblastoma cells after exposure to hypoxia (Fig. 3e). To confirm the essential role of HIF-1α in NOX4-induced FOXM1 expression, we knocked down the expression of HIF-1α with a specific siRNA. Western blot analysis showed that depletion of HIF-1α significantly inhibited NOX4-induced FOXM1 expression (Fig. 3f). To identify FOXM1 transcriptional activity in hypoxic glioblastoma cells, we focused on and investigated the protein expression of PLK1 and cyclin B1, two well-known downstream targets of FOXM1. We observed that the protein levels of cyclin B1 and cyclin D1 increased in hypoxic cells (Fig. 3g), which supported the view that HIF-1α may regulate FOXM1 expression at the transcriptional level. To determine whether HIF-1α directly regulates transcription, we assessed the impact of HIF-1α on FOXM1 transcriptional activity as reflected by a luciferase reporter assay. We observed that HIF-1α positively regulated FOXM1 transcriptional activity, reflecting a positive role of HIF-1α in FOXM1 pathway regulation (Fig. 3h-i). These data indicate that HIF-1α is necessary for NOX4 to regulate FOXM1 promoter activity. Overall, our results suggest that NOX4-mediated HIF-1α stabilization promotes FOXM1 expression.

### NOX4 and FOXM1 regulate aerobic glycolysis and proliferation in glioblastoma cells

**Fig. 4:**
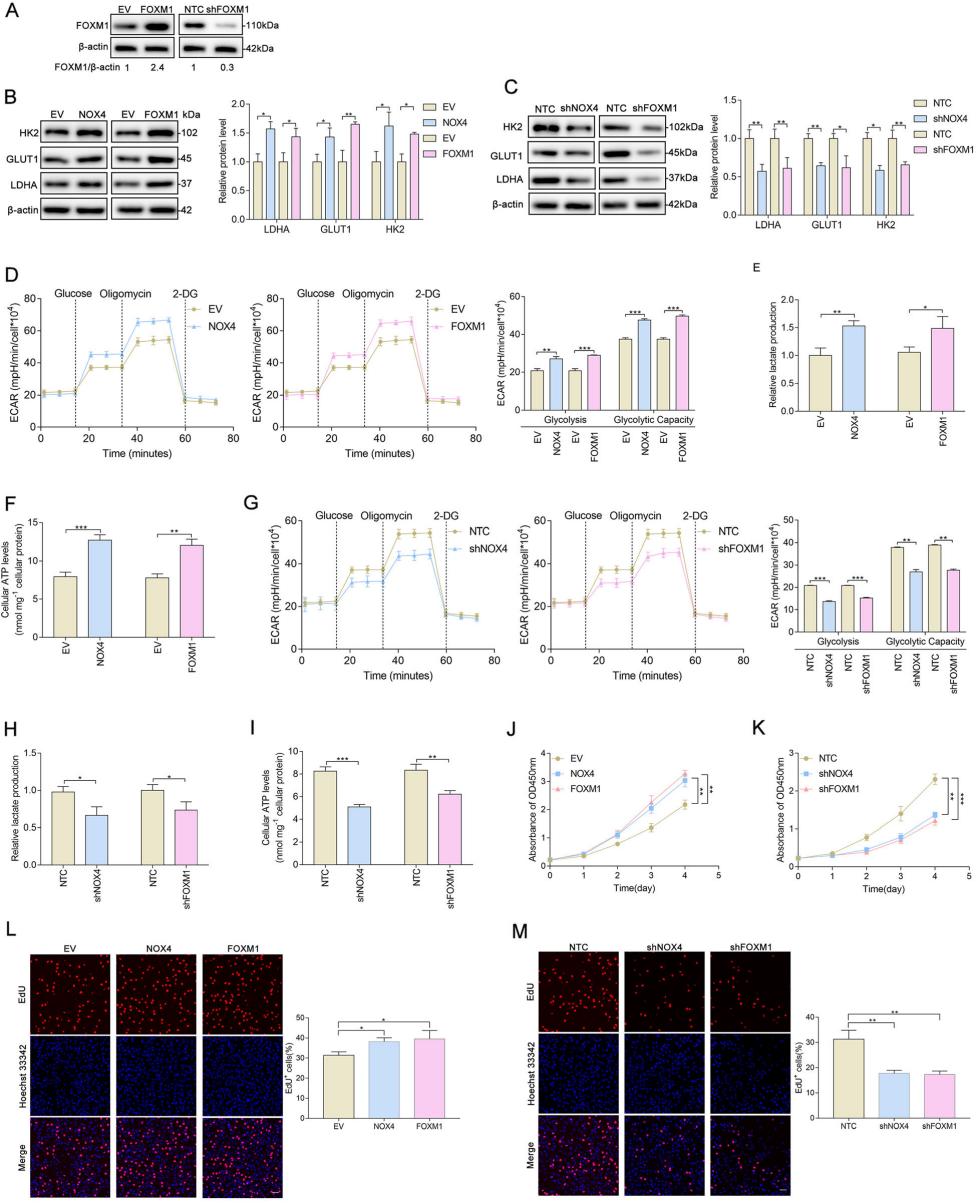
NOX4 and FOXM1 regulate aerobic glycolysis and proliferation in glioblastoma cells. **A** Western blot analysis showing the overexpression and silencing efficacy of FOXM1. **B** Western blot analysis showing that NOX4 or FOXM1 overexpression increased the protein levels of the glycolytic genes LDHA, GLUT1, and HK2. **C** Western blot analysis showing that NOX4 or FOXM1 knockdown decreased the protein levels of the glycolytic genes HK2, GLUT1, and LDHA. **D** ECAR was measured in NOX4- or FOXM1-overexpressing U87MG cells using an XFe96 Extracellular Flux Analyzer. Glycolysis and glycolytic capacity were increased in NOX4- or FOXM1-overexpressing cells. **E**-**F** NOX4 or FOXM1 overexpression increased lactate production and cellular ATP levels. **G** ECAR was measured in NOX4- or FOXM1-knockdown U87MG cells using an XFe96 Extracellular Flux Analyzer. Glycolysis and glycolytic capacity were decreased in NOX4- or FOXM1-knockdown cells. **H**-**I** NOX4 or FOXM1 knockdown decreased lactate production and cellular ATP levels. **J**-**K** The viability of U87MG cells was increased upon NOX4 or FOXM1 overexpression and decreased upon NOX4 or FOXM1 knockdown, as determined with CCK-8. **L**-**M** NOX4 or FOXM1 overexpression increased the proliferation rate of U87MG cells, whereas NOX4 or FOXM1 knockdown with specific shRNA decreased the proliferation rate of U87MG cells, as shown by EdU staining. *, *P* < 0.05; **, *P* < 0.01; ***, *P* < 0.001

Given that higher expression of NOX4 and FOXM1 predicted a worse prognosis in glioma, and based on reports that NOX4 promotes cell glycolysis in a variety of human malignancies, including glioblastoma [20], we explored the impact of NOX4 and FOXM1 on aerobic glycolysis in glioblastoma cells. First, we established stable overexpression of FOXM1 and silenced FOXM1 in U87MG cells (Fig. 4a). To confirm the roles of NOX4 and FOXM1 in the regulation of glycolytic genes, we examined the expression status of LDHA, GLUT1, and HK2 in U87MG cells. Western blot analysis showed that the levels of glycolytic genes were significantly increased in U87MG cells overexpressing either NOX4 or FOXM1 (Fig. 4b); however, the levels of these genes were decreased when NOX4 or FOXM1 was deleted (Fig. 4c). Then, we subsequently investigated whether NOX4 or FOXM1 was capable of impacting the metabolic phenotypes of U87MG cells. Notably, we found that elevated expression of NOX4 or FOXM1 significantly increased glycolysis, glycolytic capacity (Fig. 4d), lactate production (Fig. 4e), and cellular ATP levels (Fig. 4f). In contrast, the stable expression of shRNAs targeting NOX4 or FOXM1 markedly reduced glycolysis, glycolytic capacity (Fig. 4g), lactate production (Fig. 4h), and cellular ATP levels (Fig. 4i). We further investigated whether NOX4 and FOXM1 influence glioblastoma proliferation. Cell proliferation was evaluated by CCK-8 and EdU assays. NOX4 or FOXM1 overexpression significantly increased cell viability (Fig. 4j) and the percentage of EdU-positive (Fig. 4l) U87MG cells, reflecting a higher proportion of cells entering the DNA replication phase of the cell cycle, while NOX4 or FOXM1 knockdown strikingly inhibited cell viability (Fig. 4k) and markedly reduced the percentage of EdU-positive cells (Fig. 4m). Collectively, from both gain-of-function and loss-of-function studies, our data suggest that both NOX4 and FOXM1 play essential roles in regulating aerobic glycolysis and proliferation in glioblastoma cells.

### FOXM1 is critical for NOX4-induced aerobic glycolysis and proliferation in glioblastoma cells

**Fig. 5:**
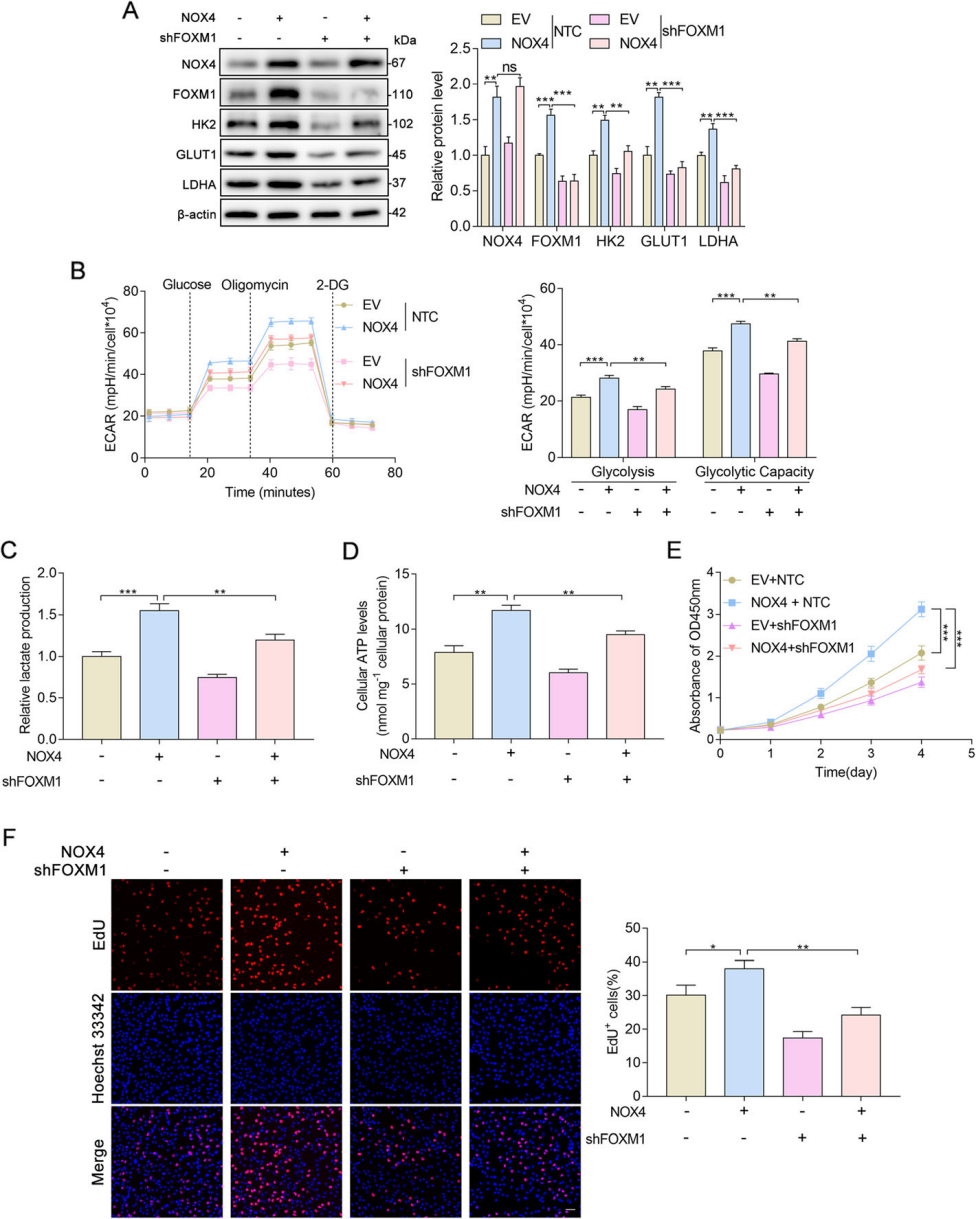
FOXM1 is critical for NOX4-induced aerobic glycolysis and proliferation in glioblastoma cells. **A** Western blot analysis showing the protein expression of the indicated genes in U87MG cells. **B** U87MG cells were infected with NOX4 overexpression (NOX4) plasmid and FOXM1 knockdown (shFOXM1) lentiviral vectors before plating in a Seahorse XFe96 analyzer and assessment with a glycolysis stress test. **C**-**D** Overexpression of NOX4 promoted the production of lactate and cellular ATP, while knockdown of FOXM1 reversed these effects, in U87MG cells. **E**-**F** CCK-8 and EdU assays showing the proliferation of the indicated U87MG cells stably expressing NOX4 + shFOXM1. *, *P* < 0.05; **, *P* < 0.01; ***, *P* < 0.001

Since NOX4 and FOXM1 regulate aerobic glycolysis in glioblastoma cells, we asked whether NOX4 promotes the Warburg effect by upregulating FOXM1 expression. To test whether FOXM1 is required for NOX4-induced aerobic glycolysis, shRNA-mediated repression of FOXM1 was performed in NOX4-overexpressing U87MG cells. We first detected the protein expression of important glycolytic enzymes involved in glucose metabolism in the indicated U87MG cells. Interestingly, we found that FOXM1 and the glycolytic enzymes LDHA, GLUT1, and HK2 were increased in NOX4 + NTC cells relative to EV + NTC U87MG cells, but this was abrogated by downregulation of FOXM1 (NOX4 + shFOXM1) (Fig. 5a). We next sought to determine whether FOXM1 was involved in elevated aerobic glycolysis regulated by NOX4. Our results revealed that the induction of glycolysis phenotypes caused by overexpression of NOX4 was obviously attenuated by downregulation of FOXM1, as shown by ECAR (Fig. 5b), production of lactate (Fig. 5c), and intracellular ATP levels (Fig. 5d), establishing FOXM1 as a functional downstream target of NOX4 in the regulation of glycolytic metabolism. Since our results clearly demonstrated that NOX4 regulates aerobic glycolysis in glioblastoma cells via FOXM1 and that increased glucose metabolism is advantageous for in proliferating cells [21], we further explored whether FOXM1 is critical for NOX4-regulated cancer proliferation. We analyzed the cell proliferation rate with CCK-8 and EdU assays and found that knockdown of FOXM1 diminished the cell proliferation-promoting effect of NOX4 (Fig. 5e, f). Taken together, these results demonstrate the critical role of FOXM1 in the induction of aerobic glycolysis and proliferation caused by constitutive NOX4 overproduction.

### Altered NOX4-FOXM1 signaling modulates tumorigenesis in vivo

**Fig. 6:**
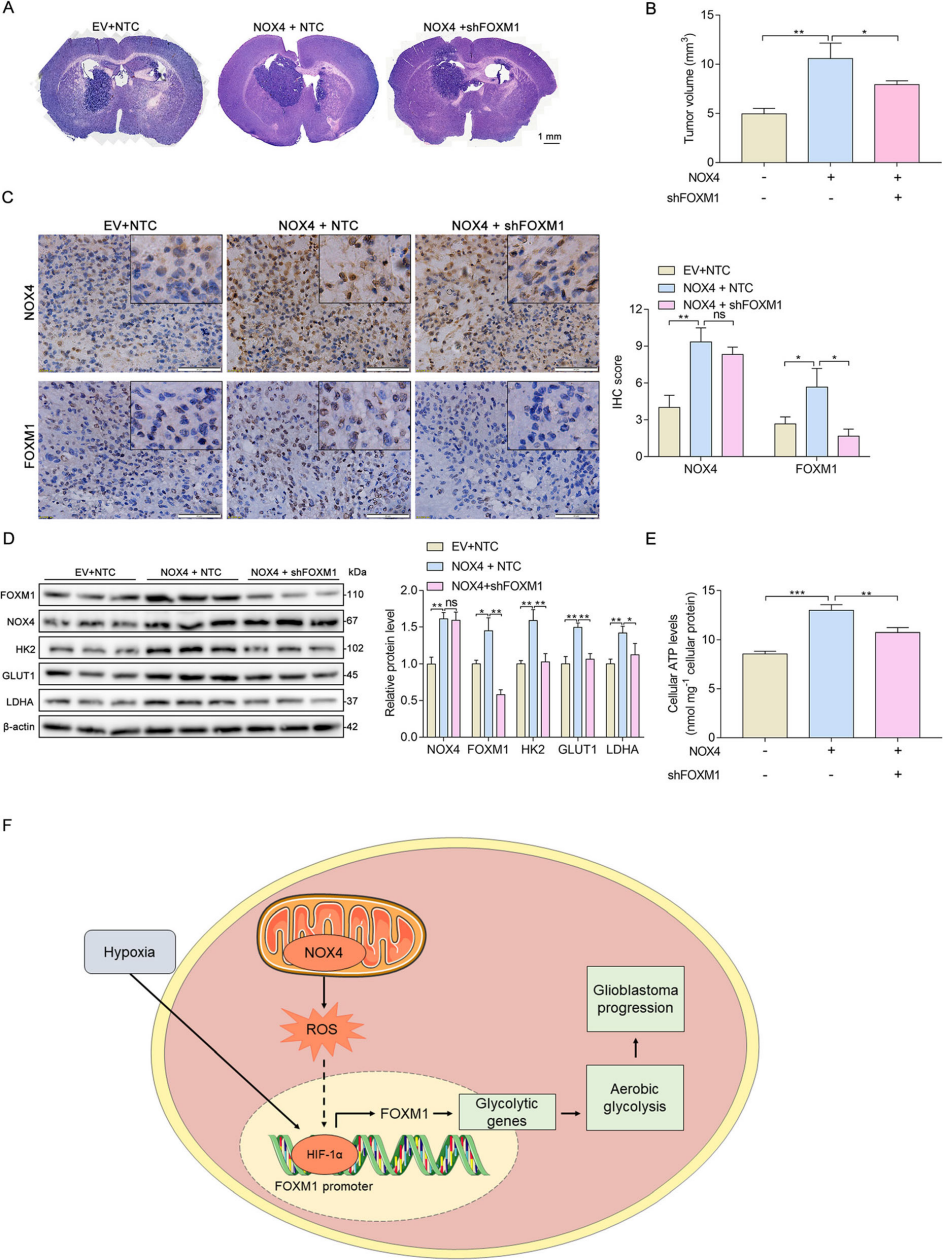
Altered NOX4-FOXM1 signaling modulates tumorigenesis in vivo. A H&E staining of the sections of xenograft mouse brains. Scale bar, 1 mm. B Tumor size (mm^3^) was measured. C-D IHC and Western blot analysis of the indicated proteins in tumor tissue samples from the indicated groups. Scale bar, 20 μm. E Cellular ATP levels were determined in tumors from xenograft mice. F Schematic model of NOX4-FOXM1 signaling-regulated aerobic glycolysis and progression in glioblastoma cells. In glioblastoma cells, NOX4-generated mitochondrial ROS mediate HIF-1α stabilization. Stabilized HIF-1α can directly bind to the FOXM1 promoter and subsequently promote the expression of FOXM1, resulting in aerobic glycolysis and progression. *, *P* < 0.05; **, *P* < 0.01; ***, *P* < 0.001

To further determine the effect of altered NOX4-FOXM1 signaling on glioblastoma biology, we extended our investigation to experiments in vivo*.* We intracranially injected nude mice with U87MG cells stably expressing EV + NTC, NOX4 + NTC, and NOX4 + shFOXM1. Compared to that in the control group, the tumor size of the NOX4 + NTC group was significantly larger, but this effect was markedly abrogated by FOXM1 knockdown (NOX4 + shFOXM1 group) (Fig. 6a, b). The protein levels of FOXM1 and aerobic glycolysis enzymes were dramatically increased in the NOX4 + NTC group, whereas knockdown of FOXM1 reversed the expression of these genes (Fig. 6c, d). Moreover, intracellular ATP levels in NOX4-overexpressing tumors were much higher, and FOXM1 depletion restored this effect (Fig. 6e). Based on these observations, we propose that NOX4-FOXM1 signaling facilitates aerobic glycolysis in glioblastoma to promote cancer progression in vivo.

## Discussion

Despite progress in the treatment and prognosis of glioblastoma, no significant progress has been made in improving survival; therefore, there is an urgent need for a better understanding of the biology of glioblastoma [1]. According to a recent study, aerobic glycolysis is regarded as one of the hallmarks of human cancer and plays an important role in tumorigenesis, and targeting metabolic changes could provide innovative possibilities to inhibit the progression of cancer [22]. In glioblastoma, astrocytes more often use aerobic glycolysis rather than oxidative phosphorylation to generate ATP regardless of oxygen availability because this process readily produces lactate and energy for proper neuronal function [23]. Thus, understanding why glioblastoma cells require metabolic changes will provide novel insights for targeted therapy. By analyzing samples from the TCGA database and clinical specimens, our data demonstrated that elevated expression of NOX4 and FOXM1 predicted a worse prognosis of glioblastoma. Mechanistic research indicated that NOX4 stimulates FOXM1 expression by increasing mitochondrial ROS to stabilize HIF-1α. Furthermore, NOX4 promoted aerobic glycolysis by positively regulating FOXM1, a transcription factor that regulates metabolic genes. We also showed that FOXM1 strongly promoted aerobic glycolysis, which has seldom been discussed in glioblastoma before.

FOXM1 is a transcription factor with important roles in cancer cell proliferation, migration, and invasion. Numerous pieces of evidence have demonstrated FOXM1 as a critical hub gene that transduces upstream signals to downstream effectors [13]. Recently, increasing evidence has implicated elevated FOXM1 expression in a variety of tumor types and correlates with tumorigenicity and a poor prognosis, including glioblastoma multiforme [24]. However, upstream regulators of FOXM1 expression have yet to be well elucidated. Thus, identifying the regulatory mechanism that underlies FOXM1 upregulation is important for further understanding the tumorigenic process and enabling the development of new strategies for tumor prevention and therapy. A previous study demonstrated that FOXM1 is activated by an inflammatory microenvironment and that an increase in FOXM1 expression promotes cancer cell proliferation and resistance to apoptosis [15]. Meanwhile, the expression of FOXM1 is also regulated by oncogenic Ras-mediated ROS generation, and elevated FOXM controls the oxidative stress balance to accelerate the growth and survival of cancer cells [25]. However, whether FOXM1 can be regulated by NOX4-derived MitoROS has not been previously explored. Considering that FOXM1 expression is positively correlated with NOX4 expression in glioma, it is speculated that NOX4 may be involved in the regulation of FOXM1 expression. To test this hypothesis, we investigated the roles of NOX4 and FOXM1 and their relationship in glioblastoma cells with in vitro and in vivo experiments. In the present study, we found that NOX4 significantly stimulated FOXM1 expression by increasing mitochondrial ROS production.

Recent studies have shown that mitochondria and NADPH oxidases are two major producers of intracellular ROS in cancer and that crosstalk exists between these two inducers [8]. NOX-generated ROS act as second messengers that are highly diffusible and induce a variety of biological effects by stimulating the expression of downstream genes [26]. In glioblastoma, NOX4 is the most frequent NOX isoform, and aberrant ROS generation of NOX4 contributes to cell proliferation and survival [17, 27, 28]. To investigate the relationship between NOX4, ROS production, and FOXM1 expression, hydrogen peroxide was used to simulate special oxidative stress, and antioxidants, NAC and Mito-Tempo were applied to scavenge ROS. Western blot analysis showed that increased ROS levels can activate FOXM1 expression and that inhibiting ROS generation significantly reduced the expression of FOXM1. These findings provide new insight and evidence that the NOX4/ROS pathway is involved in the upregulation of FOXM1 expression in glioblastoma cells. As a master regulator driving cancer progression, HIF-1α activates the transcription of a large battery of genes encoding proteins that promote multiple steps of this process, including angiogenesis, metabolism, epithelial–mesenchymal transition, and immune evasion [17, 19]. Because intratumoral hypoxia is a common feature of solid tumors, particularly in glioblastoma [29], and HIF-1α is downstream of NOX4-derived ROS [10], we clearly elucidated that NOX4-derived MitoROS induced FOXM1 overexpression by stabilizing HIF-1α.

Previous studies have indicated that FOXM1 promotes aerobic glycolysis in some cancers by directly targeting metabolic enzymes at the transcriptional level. In pancreatic cancer, FOXM1 promotes aerobic glycolysis and progression by binding directly to the LDHA promoter region and regulating the expression of the LDHA gene [16]. In hepatocellular carcinoma, FOXM1 regulates aerobic glycolysis by transactivating GLUT1 expression [30]. However, the roles of FOXM1 in metabolic changes in glioblastoma have yet to be fully elucidated. We demonstrated for the first time that FOXM1 contributed to aerobic glycolysis in glioblastoma and that this effect was regulated by NOX4-derived MitoROS generation, extending the role of FOXM1 as a master regulator of cancer metabolism. As a transcription factor, FOXM1 can regulate the transcriptional activation of downstream genes. In our study, we found that the protein levels of LDHA, GLUT, and HK2, which are the key enzymes of aerobic glycolysis, were regulated by FOXM1. It should be noted that HIF1α plays a central role in metabolic reprogramming and transcriptionally regulates glycolytic enzymes [19]; therefore, further experiments are required to validate the roles of HIF-1α and FOXM1 interaction in the modulation of metabolic changes. Although we investigated the regulatory role of FOXM1 in aerobic glycolysis in glioblastoma cells, more studies are needed to explore whether FOXM1 regulates other glycolysis enzymes, and the underlying mechanism should be elucidated.

## Conclusions

In summary, we identified for the first time that FOXM1 is a novel downstream target of NOX4-derived MitoROS, which is required for NOX4-derived MitoROS-induced aerobic glycolysis and progression in glioblastoma (Fig. 6f). Our results suggest that NOX4-FOXM1 signaling is a promising molecular target for potential therapeutic strategies for the treatment of glioblastoma.

## Supplementary Information

**Additional file 1.**

## Data Availability

Not applicable.

## Abbreviations

NOX4: NADPH oxidase 4
FOXM1: Forkhead box M1
ROS: Reactive oxygen species
HIF-1α: Hypoxia-inducible factor 1α
GBM: Glioblastoma multiforme
GLUT1: Glucose transporter 1
LDHA: Lactate dehydrogenase A
HK2: Hexokinase 2
ECAR: Extracellular acidification rate
NAC: N-acetyl-cysteine
ATP: Adenosine triphosphate
