# Supplementary figures and images for: NOX4-derived ROS-induced overexpression of FOXM1 regulates aerobic glycolysis in glioblastoma
Source: BMC Cancer. 2021 Nov 5;21:1181. doi: 10.1186/s12885-021-08933-y (PMC8571893; doi:10.1186/s12885-021-08933-y)

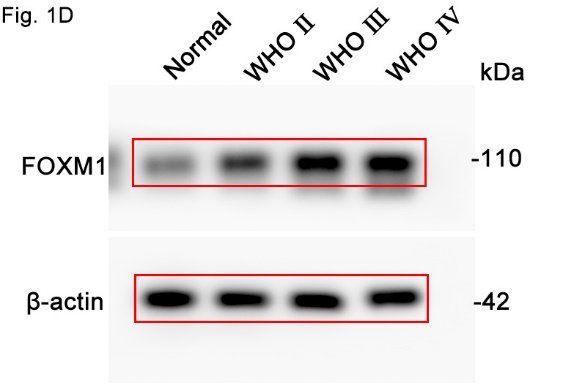

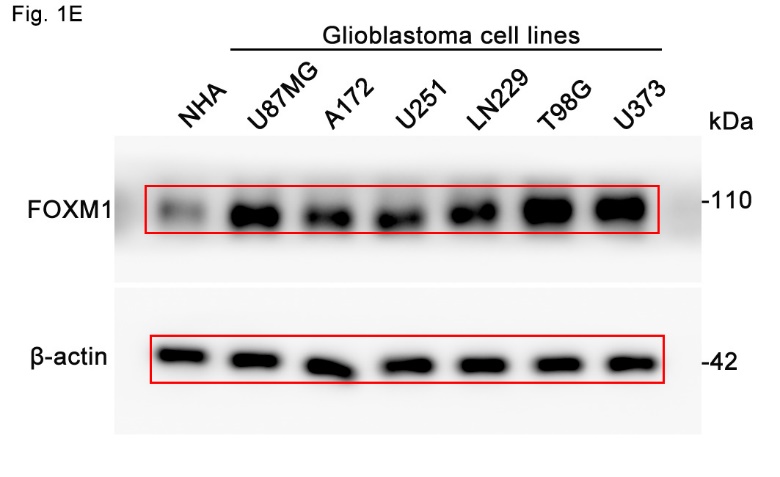

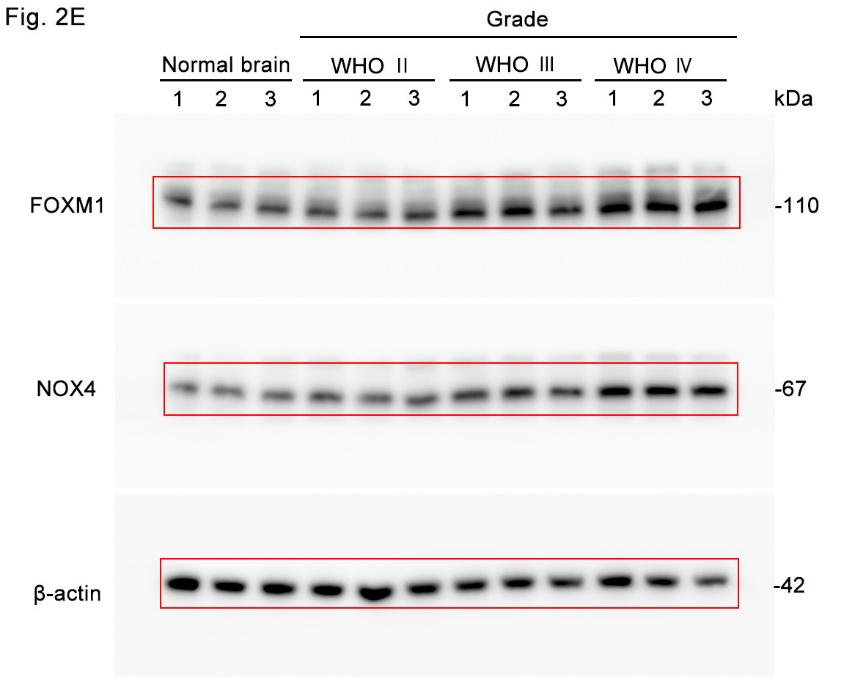

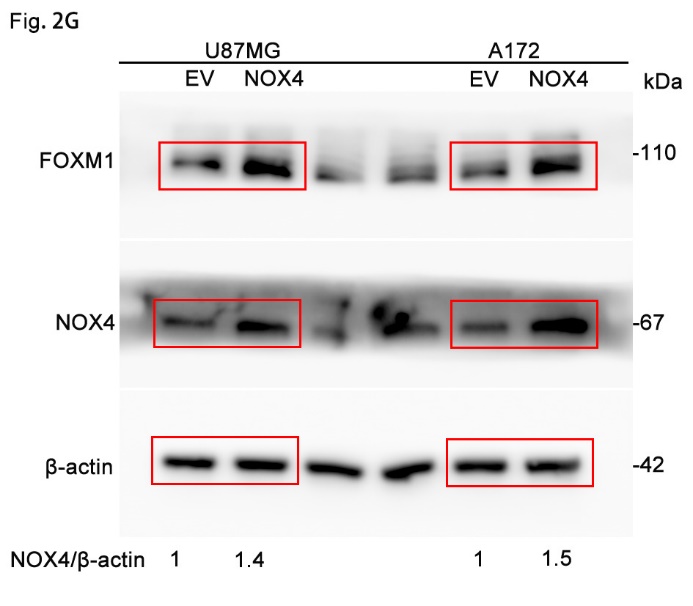

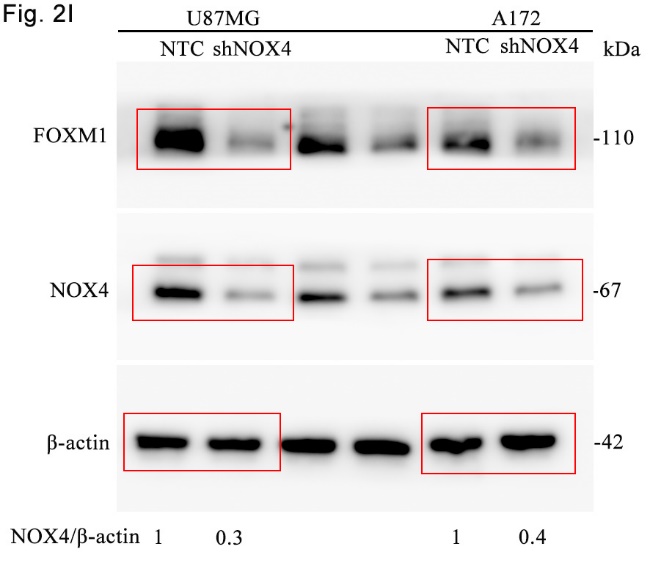

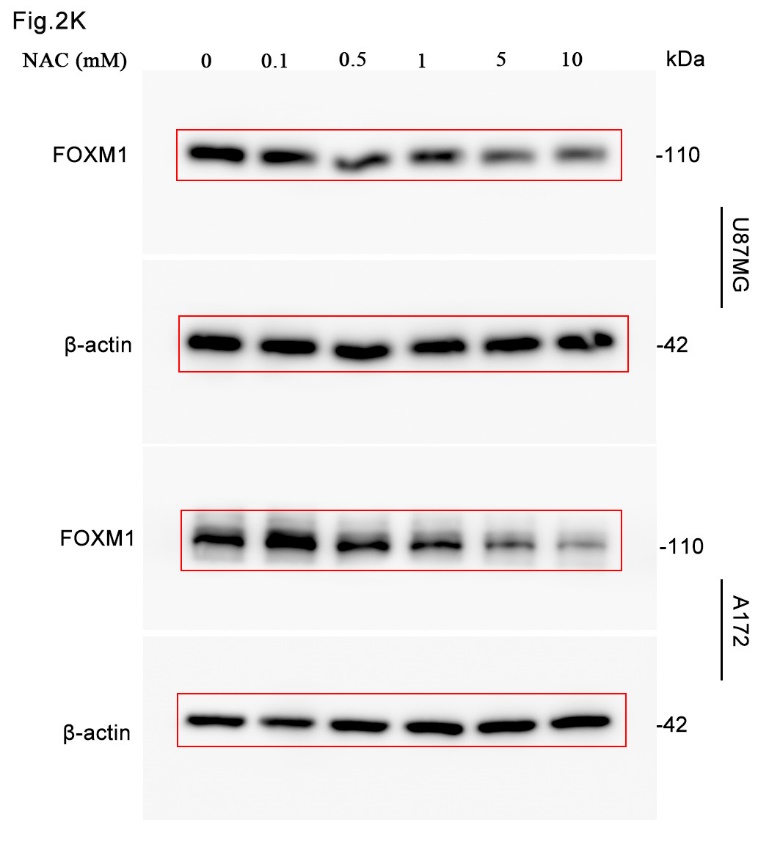

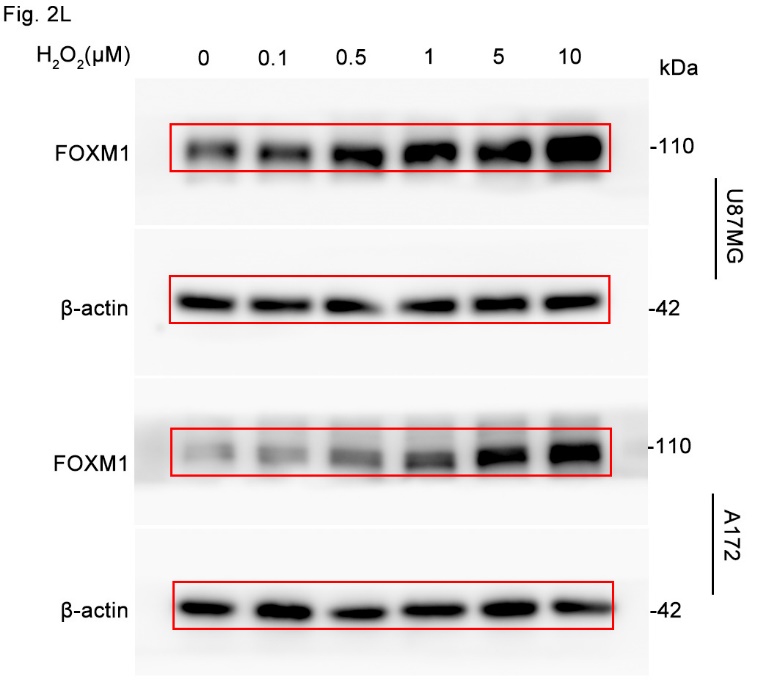

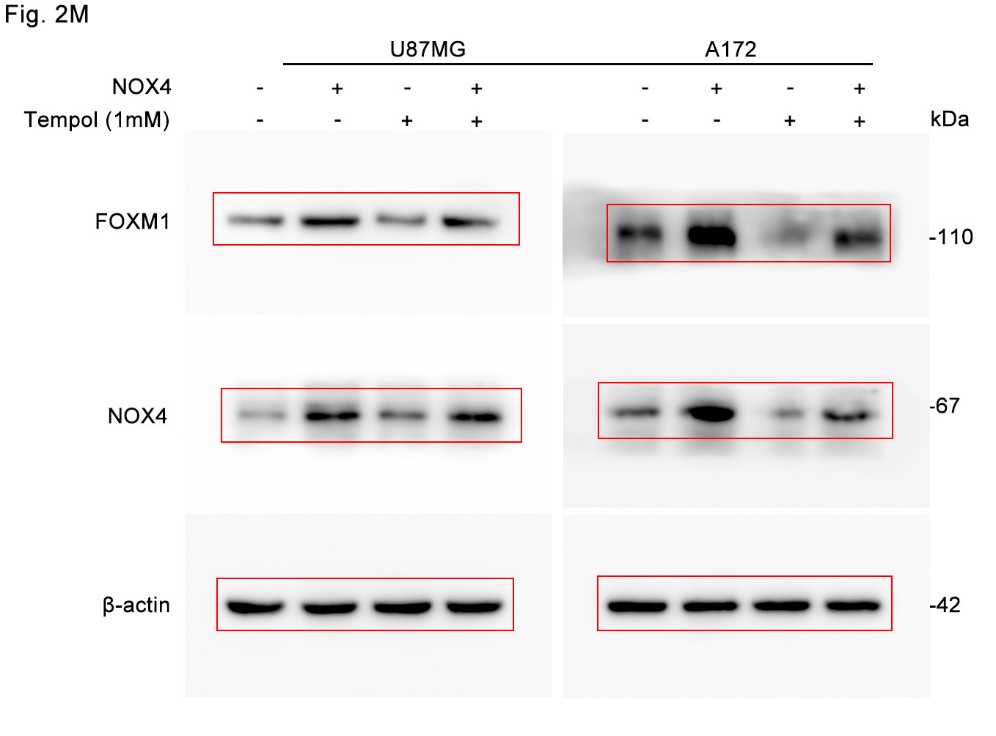

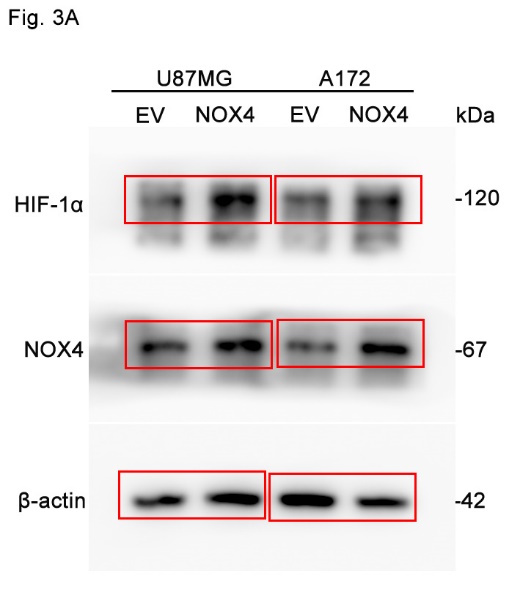

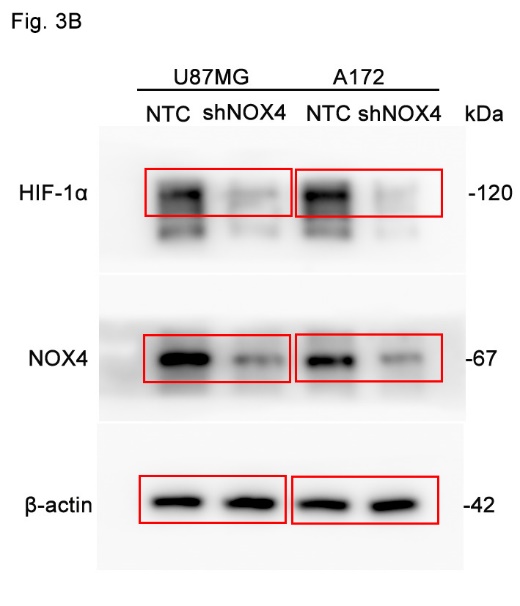

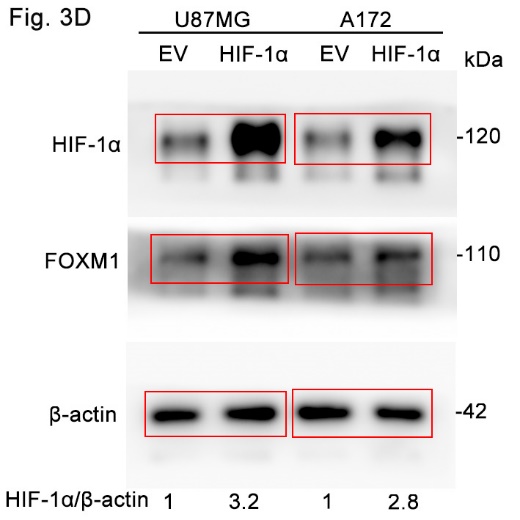

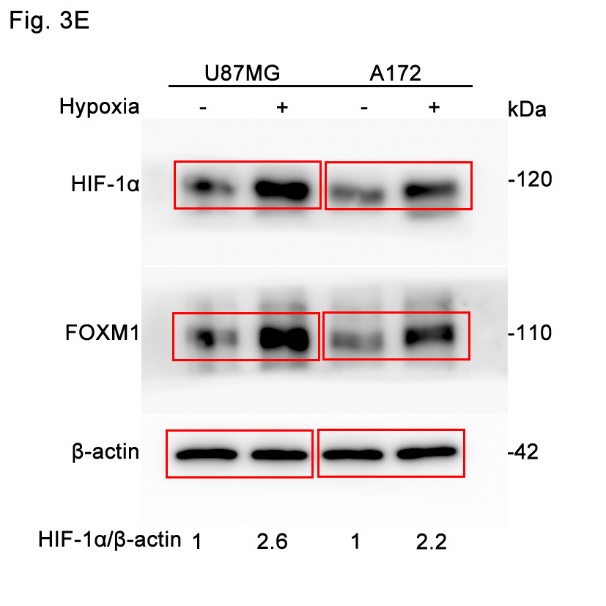

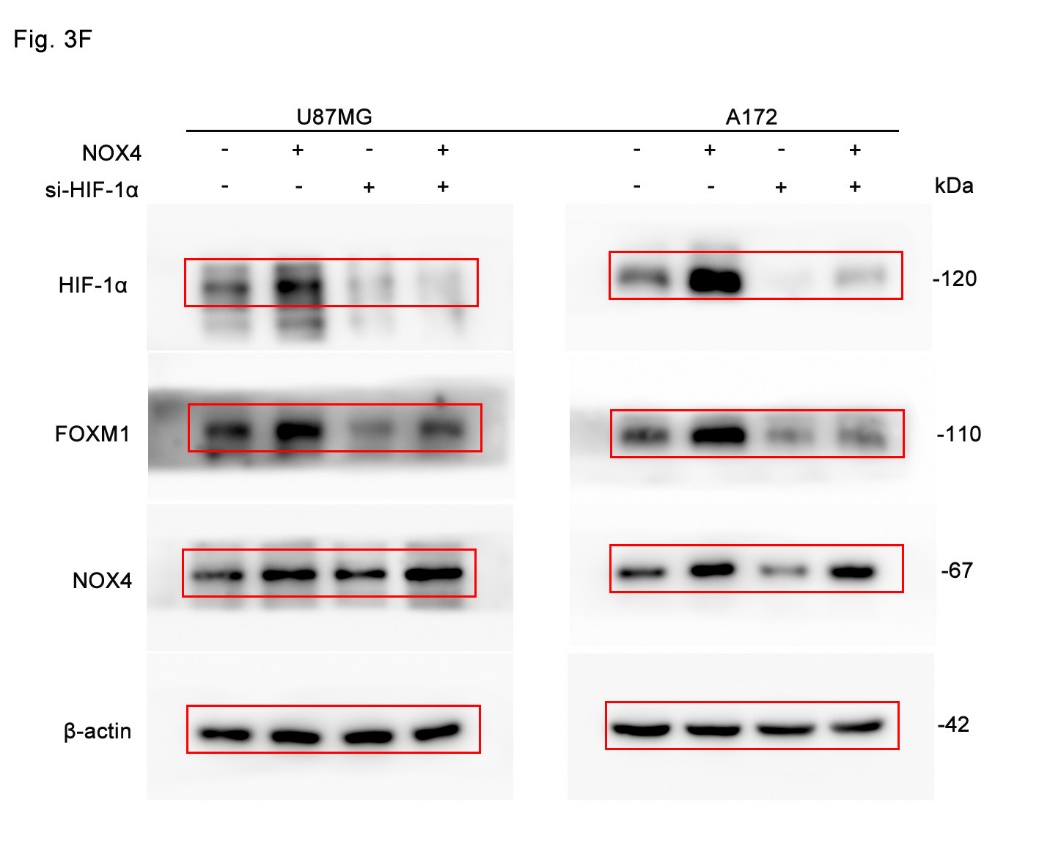

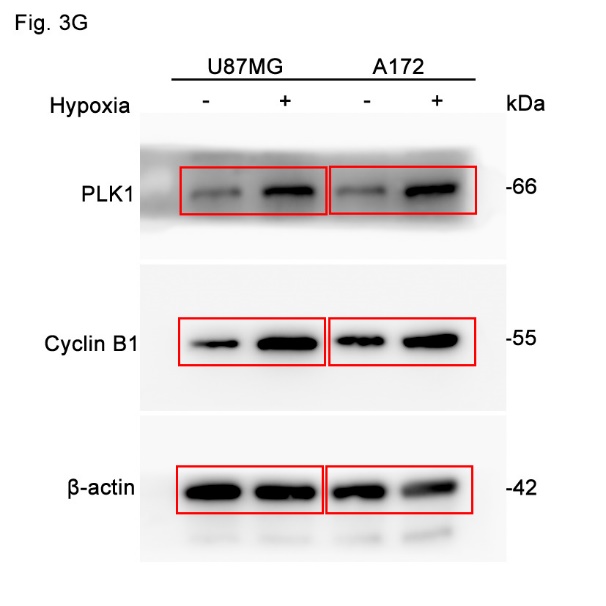

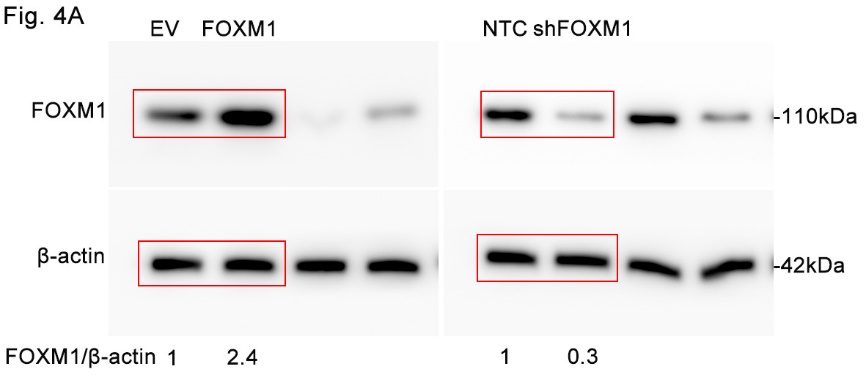

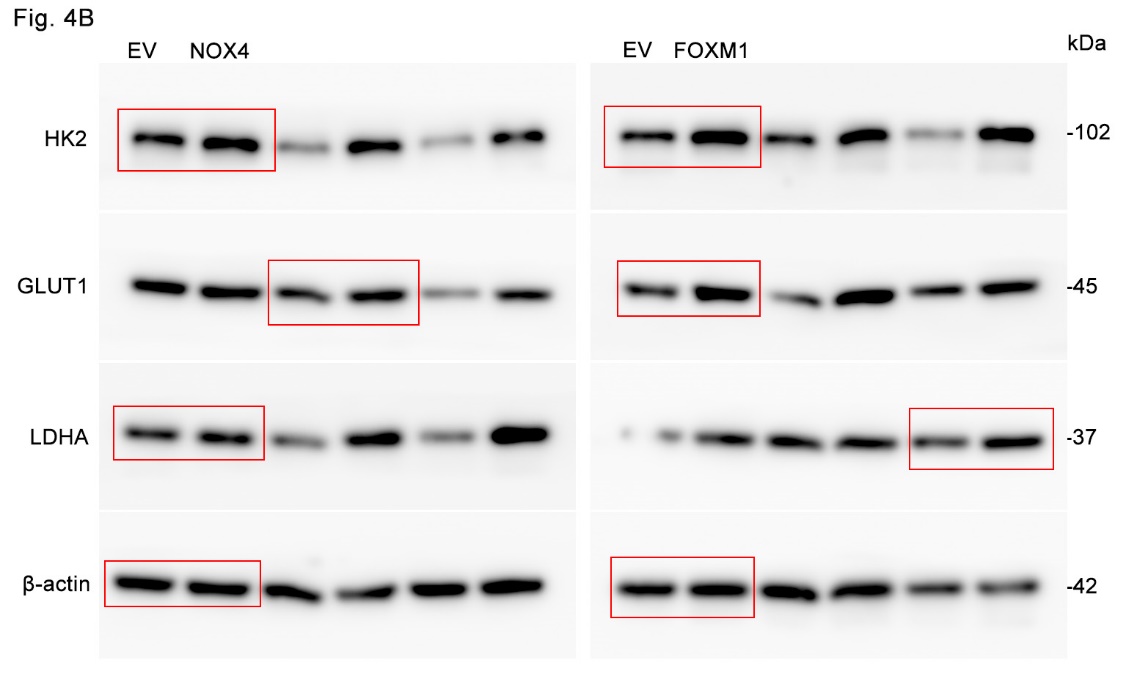

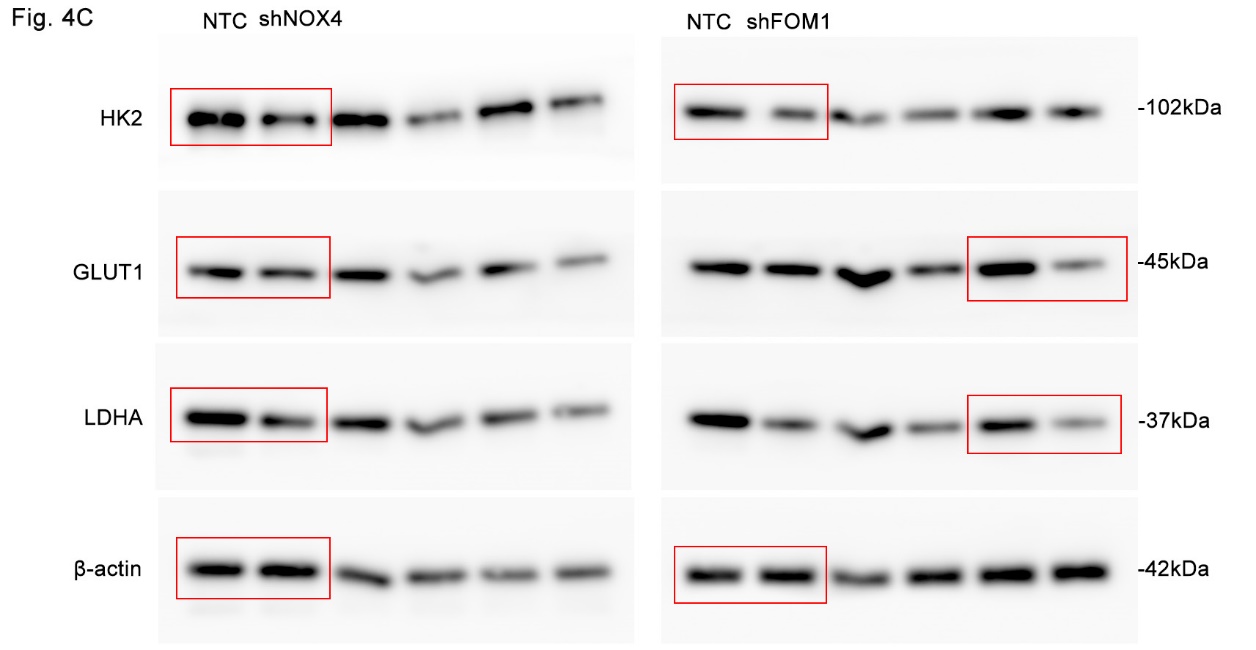

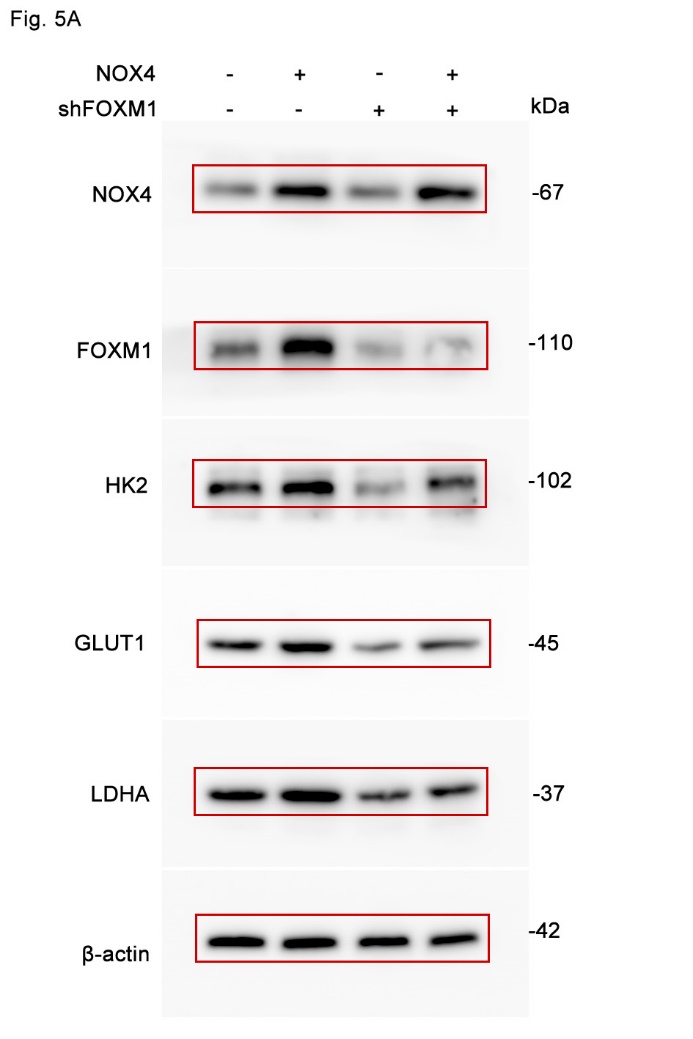

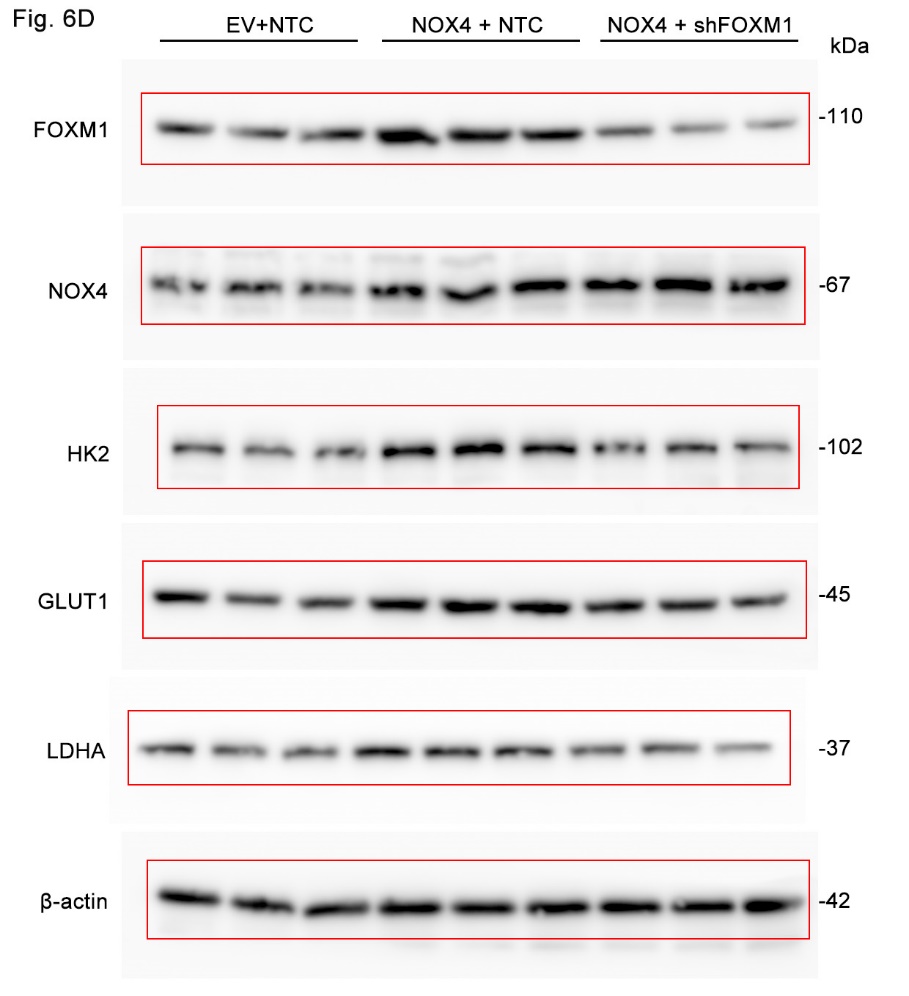

Supplement: Supplementary file 1 — Additional file 1. [file 12885_2021_8933_MOESM1_ESM.docx]
